# Pathogenic Gene Mutations or Variants Identified by Targeted Gene Sequencing in Adults With Hemophagocytic Lymphohistiocytosis

**DOI:** 10.3389/fimmu.2019.00395

**Published:** 2019-03-07

**Authors:** Yi Miao, Hua-Yuan Zhu, Chun Qiao, Yi Xia, Yiling Kong, Yi-Xin Zou, Yu-Qing Miao, Xiao Chen, Lei Cao, Wei Wu, Jin-Hua Liang, Jia-Zhu Wu, Li Wang, Lei Fan, Wei Xu, Jian-Yong Li

**Affiliations:** ^1^Department of Hematology, The First Affiliated Hospital of Nanjing Medical University, Jiangsu Province Hospital, Nanjing, China; ^2^Key Laboratory of Hematology, Nanjing Medical University, Nanjing, China; ^3^Collaborative Innovation Center for Cancer Personalized Medicine, Nanjing Medical University, Nanjing, China

**Keywords:** hemophagocytic lymphohistiocytosis, targeted gene sequencing, mutation, *UNC13D*, *AP3B1*, *ITK*, LYST, PRF1

## Abstract

Hemophagocytic lymphohistiocytosis (HLH) can be classified into primary HLH and secondary HLH. Primary HLH usually occurs in infants and children with an underlying genetic defect, and there are also teens and occasional adults with primary HLH. Most cases with secondary HLH are adult patients with secondary triggers including infections, malignancies, and autoimmune diseases. The distinction between primary HLH and secondary HLH seems to be less straightforward, as patients with secondary HLH may also have genetic defects while primary HLH can be triggered by secondary causes. In this study, using amplicon-based targeted gene sequencing (TGS), we sequenced eighteen HLH-related genes in 112 adult HLH cases, which were mostly secondary HLH. Mutations or rare variants were identified in 48 cases (42.9%). All the variants except one were missense variants, and biallelic gene mutations were identified in 3 cases in which only one case harbored homogenous missense mutation. Recurrent variants including *UNC13D* p.G863D and *AP3B1* p.T359A are much more prevalent in our cohort than in normal East Asian population, and *in silico* analysis predicted pathogenicity of these variants. In conclusion, according to our study, genetic defects may also contribute to the development of adult HLH cases or secondary HLH cases.

## Introduction

Hemophagocytic lymphohistiocytosis (HLH) is characterized by overwhelming inflammatory cytokine storm. Patients with HLH usually present with fever, hepatosplenomegaly and cytopenia (1). Traditionally, HLH can be classified into primary and secondary HLH according to the underlying etiologies. Primary HLH is caused by genetic defects and always present in young children, and increasing evidence suggests that primary HLH could also occur in teens and young adults (2, 3). Secondary HLH, mainly identified in adults, is mostly triggered extrinsic factors including infections, malignancies or autoimmune diseases (2). Suppressing cytokine storm using HLH-94 or HLH-2004 regimen is essential in the induction treatment of HLH, however, the subsequent treatments vary dependent on the underlying etiologies (4). For patients with primary HLH, allogeneic hematopoietic stem cell transplantation needs to be considered, while for secondary HLH, treatments focused on eliminating the secondary triggers are of vital importance (4).

In recent years, increasing evidence suggests that the distinction between primary HLH and secondary HLH may be less clear. HLH could be result of the combination of inherited genetic mutations, susceptibility loci and extrinsic triggers. The cases with so-called “secondary” HLH may also have genetic defects in cytotoxic lymphocyte function. Recently, adult cases with HLH caused by influenza A (H1N1) infection were found to have heterozygous *LYST* mutations or *PRF1* p.A91V mutation, which led to deceased NK cell cytolytic function (5). Gene mutations related to HLH were also identified in patients with macrophage activation syndrome (MAS)/HLH, which was secondary to rheumatic diseases (6, 7). The study by Zhang et al. showed that adults with HLH might also harbor missense or splice-site variants in familial HLH associated genes, however, in this study only 3 genes were studied by Sanger sequencing (3). To better understand the genetic background of adult HLH, we performed TGS of 18 genes related to HLH in a cohort of 112 adult patients with HLH.

## Methods

### Patients

This study included 112 patients diagnosed as HLH. The diagnosis of HLH was based on HLH-2004 criteria, and only cases that fulfilled 5 or more HLH-2004 criteria were diagnosed as HLH and included in our study (8). Every participant provided informed consent according to the Declaration of Helsinki. This study was approved by the Ethics Committee of the First Affiliated Hospital of Nanjing Medical University.

### Targeted Gene Sequencing

A targeted gene sequencing (TGS) panel consisting of 18 HLH-associated genes was designed (Table 1). We used genome build hg19 as reference and included all the exons in the above genes into this panel. The genomic DNA was isolated using peripheral blood or bone marrow samples using QIAamp DNA Blood Mini Kit (Qiagen). Amplification was performed with 20 ng gDNA using Ion AmpliSeq Library Kit 2.0 for each multiplex PCR reaction (Ion Torrent, Thermo Fisher Scientific). Libraries were then constructed using KAPA HyperPlus Kits (Kapa Biosystems, Wilmington, MA, USA). After purification using microbeads (Agencourt AMPure XP reagent, Beckman Coulter, Brea, CA, USA), libraries were quantified by Qubit 2.0 Fluorometer (Life Technologies) and diluted to a concentration of 1 ng/μl. The libraries were pooled and denatured, and further diluted to 12 pmol/L. The pooled libraries (600 μl) were then sequenced on Illumina Miseq sequencing platform or Illumina Hiseq sequencing platform. The median value of the mean coverage for each sample was 4,712 (range: 1,694–14,368).

**Table 1:** Genes included in targeted sequencing panel.

**Gene**	**locus**	**Number of amplicons**	**Associated diseases or related functions**
AP3B1	5q14.1	43	Hermansky-Pudlak syndrome type 2
ARF6	14q21.3	4	Involved in cytotoxic granule secretion
CD27	12p13.31	11	EBV infection including EBV-associated lymphoproliferative disease/hemophagocytic lymphohistiocytosis
CORO1A	16p11.2	13	EBV-associated B-cell lymphoproliferation
CTPS1	1p34.2	19	Immunodeficiency associated with EBV infection
GNLY	2p11.2	7	Co-localizing with perforin in the lytic granules of cytotoxic T and NK cells
GZMB	14q12	8	Co-localizing with perforin in the lytic granules of cytotoxic T and NK cells
ITK	5q33.3	20	Inducible T-cell kinase deficiency
LAMP1	13q34	14	Endosomal/lysosomal pathway
LYST	1q42.3	103	Chediak-Higashi syndrome
PRF1	10q22.1	12	Familial hemophagocytic lymphohistiocytosis type 2
RAB27A	15q21.3	5	Griscelli syndrome type 2
SH2D1A	Xq25	5	X-linked lymphoproliferative disease type 1
SRGN	10q22.1	4	Binding to granzyme B and involved in the granule mediated apoptosis
STX11	6q24.2	5	Familial hemophagocytic lymphohistiocytosis type 4
STXBP2	19p13.2	30	Familial hemophagocytic lymphohistiocytosis type 5
UNC13D	17q25.1	40	Familial hemophagocytic lymphohistiocytosis type 3
XIAP	Xq25	13	X-linked lymphoproliferative disease type 2

### Bioinformatics and Statistical Analyses

We used Burrows-Wheeler Aligner to map the sequencing reads to hg19 genome (9). Only bases that were covered by at least 50 reads were qualified for variant calling. Variant calling was performed using the SAMtools software (10). Integrative Genomics Viewer (IGV) version 2.3.32 was used for visualization of sequencing reads and visual assessment of detected variants (11). To confirm a heterozygous variant, variant alleles were required to be present in more than 15% of mapped reads. The Exome Aggregation Consortium (ExAC; http://exac.broadinstitute.org/) was referred to extract the frequencies of variants in normal Eastern Asian population. And 1,000 Genomes Project (http://www.ncbi.nlm.nih.gov/variation/tools/1000genomes/) was referred to evaluate the frequencies of variants in the whole population. Variants those with mean allele frequencies lower than 1% in normal Eastern Asia population were included into further analysis. *In silico* analysis of candidate variants was carried out using SIFT (12), Polyphen-2 (13), and MutationTaster (14).

Student *t*-test was used to compare continuous variables and fisher exact test or chi-square test was used to compare categorical variables. Kaplan-Meier method was used to construct survival curves and log-rank test was used to compare the difference. The primary endpoint in our study was survival at 8 weeks after admission to our hospital. Statistical analyses were performed using Graphpad Prism 6 (GraphPad Software, San Diego, CA) software. *P* ≤ 0.05 (2-sided) was considered statistically significant.

## Results

### Mutations or Rare Variants Identified by TGS

Totally, 112 cases were analyzed in this study, which included 66 cases of malignancy-associated HLH, 23 infection-associated HLH, 4 autoimmune disease-associated HLH as well as 19 HLH with unknown origin. Almost all the cases of malignancy-associated HLH were lymphoma related (including mature lymphoid malignancies in leukemic phase), except two cases. The median age was 41.5 and the male/female ratio was about 3:2. There are more male patients in our cohort, as most of patients are lymphoma patients (64/112, 57.1%). It is known that, in major subtypes of lymphoma, including diffuse large B cell lymphoma, peripheral T cell lymphoma and extranodal NK/T cell lymphoma, there are more male patients (15).

Forty-eight patients (48/112, 42.9%) harbored mutations or rare variants in genes studied in the TGS panel. Except for one nonsense mutation, all the mutations were missense base substitutions. One case had homozygous mutation (P6, *UNC13D* p.G863D) and two cases had compound heterozygous mutations (P38, *CORO1A* p.L235V and p.P277R; P24, *PRF1* p.R33H, and p.R4C). Three patients were double heterozygous. In cases with double heterozygous mutations, P21 had heterozygous *STX11* p.F281C mutation and *LYST* p.D2665G mutation, and *STX11* and *LYST* are both involved in the degranulation of cytotoxic cells, suggesting these two mutations may cooperate in the development of HLH. P32 had a single *ITK* p.R581W mutation and a single *STX11* p.F281C mutation. And P40 had a *CORO1A* p.A88T mutation and a *LYST* p.E1036A mutation. The remaining cases (42/48, 87.5%) carried only one mutated allele in one of these genes.

**Figure 1:**
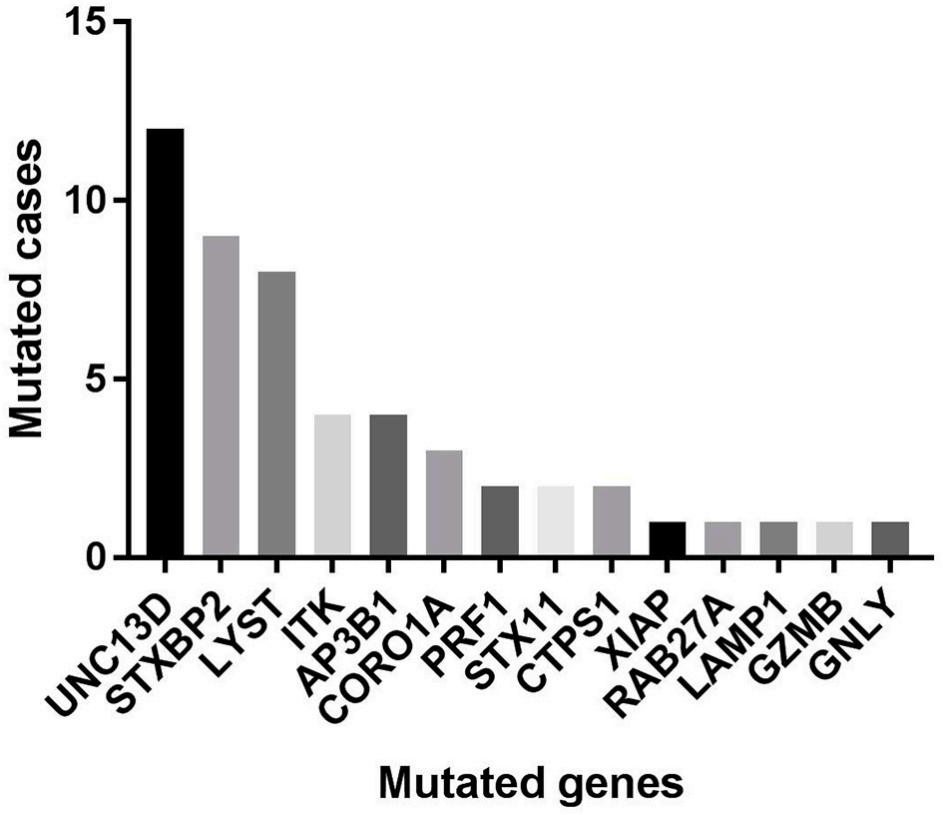
The numbers of cases with each mutated gene in our cohort.

**Figure 2:**
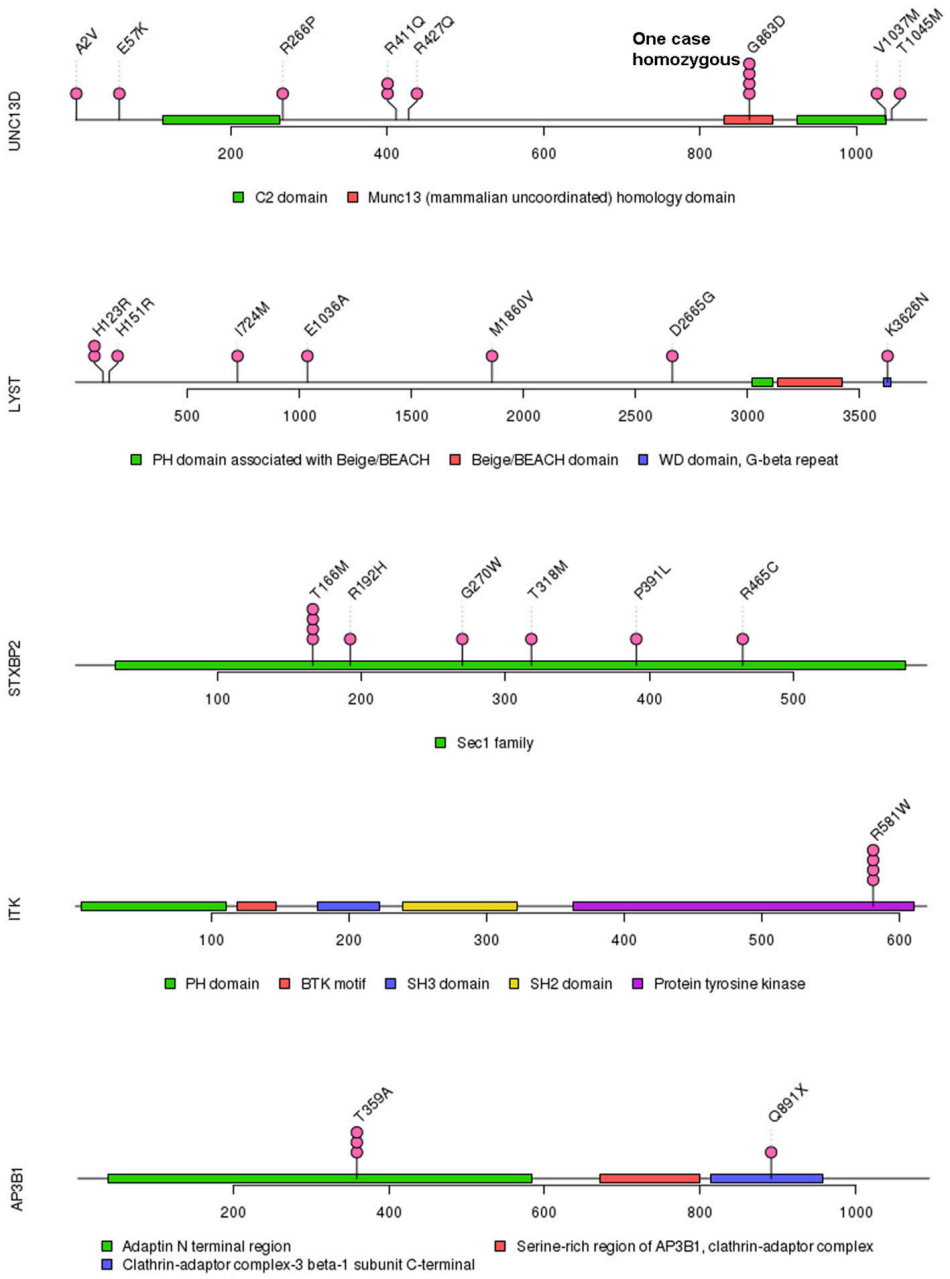
Mutations in *UNC13D, LYST, STXBP2, ITK*, and *AP3B1*.

Of 18 genes tested in our cohort, 14 genes were found to be mutated at least one time, and *UNC13D* was the most frequently mutated gene (Figure 1). Thirty-nine mutations or rare variants were identified in this cohort, and *in silico* analysis and frequencies in normal Eastern Asian population (from ExAC database) and 1,000 genomes of these variants were summarized in Table 2. Some of the variants were previously reported in cases of HLH and the references were provided in Table 2 (16–25). And the conditions and clinical significance of variants documented in ClinVar database (https://www.ncbi.nlm.nih.gov/clinvar) were also listed in Table 2. We further classified these variants based on the pathogenicity predicted by the algorithms. These variants that were predicted to be pathogenic (including possibly pathogenic) or not pathogenic by all three algorithms were classified as “pathogenic variants” (*n* = 12) or “benign variants” (*n* = 7), respectively. The remaining variants were classified as “variants of uncertain significance” (*n* = 20). The variants involving *UNC13D, LYST, STXBP2, ITK*, and *AP3B1* were depicted in Figure 2.

**Table 2:** Summary of variants detected in our cohort.

**Variants**	**Reference sequence**	**Cases**	**SIFT**	**Polyphen2**	**MutationTaster**	Frequency in normal eastern asian population*	**Frequency in 1,000 genomes**	**Literature**	**ClinVar (condition and clinical significance)**
AP3B1 p.Q891X (c.C2671T)	NM_003664	1	Not pathogenic	NA	Pathogenic	0	–	NA	No
AP3B1 p.T359A (c.A1075G)	NM_003664	3	Pathogenic	Possibly pathogenic	Pathogenic	0.0002	0.0001997	(16)	No
CORO1A p.A88T (c.G262A)	NM_007074	1	Not pathogenic	Pathogenic	Pathogenic	–	–	NA	No
CORO1A p.R199H (c.G596A)	NM_007074	1	Pathogenic	Not pathogenic	Polymorphism	–	–	NA	No
CORO1A:p.L235V (c.C703G)	NM_007074	1	Not pathogenic	Not pathogenic	Pathogenic	–	–	NA	No
CORO1A:p.P277R (c.C830G)	NM_007074	1	Pathogenic	Not pathogenic	Pathogenic	–	–	NA	No
CTPS1 p.K460R (c.A1379G)	NM_001905	1	Not pathogenic	Not pathogenic	Pathogenic	0.0005	0.0003994	NA	No
CTPS1 p.M283V (c.A847G)	NM_001905	1	Not pathogenic	Not pathogenic	Pathogenic	0.0005	–	NA	No
GNLY:p.H35D (c.C103G)	NM_006433	1	Not pathogenic	Not pathogenic	Polymorphism	0.0003	0.000399	NA	No
GZMB p.R120Q (c.G359A)	NM_006433	1	Not pathogenic	Not pathogenic	Polymorphism	0	0.0003994	NA	No
ITK p.R581W (c.C1741T)	NM_005546	4	Pathogenic	Pathogenic	Pathogenic	0.0081	0.0009984	(17)	Conditions: lymphoproliferative syndrome 1, lymphoproliferative syndrome; clinical significance: conflicting interpretations of pathogenicity
LAMP1 p.P196L (c.C587T)	NM_005561	1	Pathogenic	Pathogenic	Polymorphism	–	–	NA	No
LYST p.D2665G (c.A7994G)	NM_000081	1	Not pathogenic	Possibly pathogenic	Pathogenic	0.0009	0.0009984	NA	No
LYST p.E1036A (c.A3107C)	NM_000081	1	Not pathogenic	Not pathogenic	Polymorphism	0.0008	0.0001997	NA	No
LYST p.H123R (c.A368G)	NM_000081	2	Not pathogenic	Possibly pathogenic	Polymorphism	0.0095	0.0021965	(18)	Conditions: Chédiak-Higashi syndrome, not specified; clinical significance: conflicting interpretations of pathogenicity
LYST p.H151R (c.A452G)	NM_000081	1	Pathogenic	Pathogenic	Pathogenic	0.0009	0.0003994	(17, 19)	No
LYST p.I724M (c.A2172G)	NM_000081	1	Not pathogenic	Possibly pathogenic	Polymorphism	0.0006	–	NA	No
LYST p.K3626N (c.A10878T)	NM_000081	1	Not pathogenic	Pathogenic	Pathogenic	–	–	NA	No
LYST p.M1860V (c.A5578G)	NM_000081	1	Pathogenic	Not pathogenic	Pathogenic	–	–	NA	No
PRF1 p.A196P (c.G586C)	NM_001083116	1	Pathogenic	Pathogenic	Pathogenic	–	–	NA	No
PRF1 p.R33H (c.G98A)	NM_001083116	1	Pathogenic	Not pathogenic	Polymorphism	0.0002	0.0001997	(3, 20–22)	No
PRF1 p.R4C (c.C10T)	NM_001083116	1	Not pathogenic	Not pathogenic	Polymorphism	0.008	0.0027955	(3, 22)	Conditions: aplastic anemia, hemophagocytic lymphohistiocytosis, familial, 2, familial hemophagocytic lymphohistiocytosis, not specified, not provided; clinical significance: conflicting interpretations of pathogenicity
RAB27A p.T85M (c.C254T)	NM_004580	1	Pathogenic	Pathogenic	Pathogenic	0.0001	–	NA	No
STX11 p.F281C (c.T842G)	NM_003764	2	Not pathogenic	Not pathogenic	Polymorphism	0.0001	0.0001997	NA	No
STXBP2 p.G270W (c.G808T)	NM_006949	1	Pathogenic	Pathogenic	Pathogenic	0.0005	0.0003994	NA	No
STXBP2 p.P391L (c.C1172T)	NM_006949	1	Pathogenic	Pathogenic	Pathogenic	–	–	NA	No
STXBP2 p.R192H (c.G575A)	NM_006949	1	Pathogenic	Pathogenic	Pathogenic	–	–	(19, 23)	No
STXBP2 p.R465C (c.C1393T)	NM_006949	1	Pathogenic	Pathogenic	Pathogenic	0	0.0001997	NA	Condition: familial hemophagocytic lymphohistiocytosis type 5; clinical significance: uncertain significance
STXBP2 p.T166M (c.C497T)	NM_006949	4	Not pathogenic	Not pathogenic	Polymorphism	0.0076	0.0025959	(23)	Condition: not specified; clinical significance: Likely benign
STXBP2 p.T318M (c.C953T)	NM_006949	1	Not pathogenic	Pathogenic	Pathogenic	0.005	0.0011981	(21)	No
UNC13D p.A2V (c.C5T)	NM_199242	1	Pathogenic	Possibly pathogenic	Polymorphism	0	–	NA	No
UNC13D p.E57K (c.G169A)	NM_199242	1	Not pathogenic	Not pathogenic	Polymorphism	0	–	NA	
UNC13D p.G863D (c.G2588A)	NM_199242	4	Pathogenic	Pathogenic	Pathogenic	0.0043	0.0013978	(17, 21, 24)	Condition: hemophagocytic lymphohistiocytosis, familial, 3; clinical significance: uncertain significance
UNC13D p.R266P (c.G797C)	NM_199242	1	Pathogenic	Pathogenic	Pathogenic	–	–	NA	No
UNC13D p.R411Q (c.G1232A)	NM_199242	2	Not pathogenic	Possibly pathogenic	Pathogenic	0.008	0.0007987	(16, 21, 25)	No
UNC13D p.R427Q (c.G1280A)	NM_199242	1	Not pathogenic	Pathogenic	Pathogenic	0.0003	0.0001997	(21, 24)	No
UNC13D p.T1045M (c.C3134T)	NM_199242	1	Not pathogenic	Possibly pathogenic	Pathogenic	0	0.0001997	(21, 24)	No
UNC13D p.V1037M (c.G3109A)	NM_199242	1	Not pathogenic	Possibly pathogenic	Pathogenic	–	–	NA	No
XIAP p.A321G (c.C962G)	NM_001167	1	Pathogenic	Pathogenic	Pathogenic	0.0046	0.0007947	NA	No

* *From the ExAC database. “–”indicated no data; NA, not available*.

Of these variants, seven variants were recurrently identified (Table 2). *UNC13D* p.G863D was identified in four patients, including one patient with homozygous mutation. The allele frequency of *UNC13D* p.G863D in our cohort was higher than that in normal East Asian population [5/224 [2.2%] vs. 37/8638 [0.4%]; odds ratio [OR] 5.307, 95%CI 2.066–13.64, *p* = 0.0007]. *In silico* analysis with three algorithms revealed that the functional consequence of *UNC13D* p.G863D missense mutation was damaging, suggesting *UNC13D* p.G863D was pathogenic in the development of HLH in these four cases. Recurrent *ITK* p.R581W mutation occurred in 4 cases, and the allele frequency of this mutation was also higher than that in normal East Asian population [4/224 [1.8%] vs. 70/8652 [0.8%], OR 2.229, 95% CI 0.8065–6.161; *p* = 0.1165], although not statistically significant. Prediction using three algorithms all suggested this amino acid substitution produced damaging effects to ITK protein. *AP3B1* p.T359A occurred more frequently in our cohort as compared with normal East Asian population [3/224 [1.4%] vs. 2/8648 [0.02%], OR 58.68, 95% CI 9.753-353.1, *p* = 0.0002], meanwhile, *in silico* analysis also confirmed the pathogenicity of this mutation. Four cases had the rare variant *STXBP2* p.T166M, and the frequency of this variant in the HLH cohort did not differ with that in normal East Asian population [4/224 [1.8%] vs. 5/660 [0.75%], OR 2.382, 95% CI 0.6338–8.951, *p* = 0.2422], and *in silico* prediction showed that this variant was benign to the function of the protein, suggesting this variant may not carry pathological significance in HLH.

### The Association of Variants With Clinical Variables and Short-Term Prognosis

**Figure 3:**
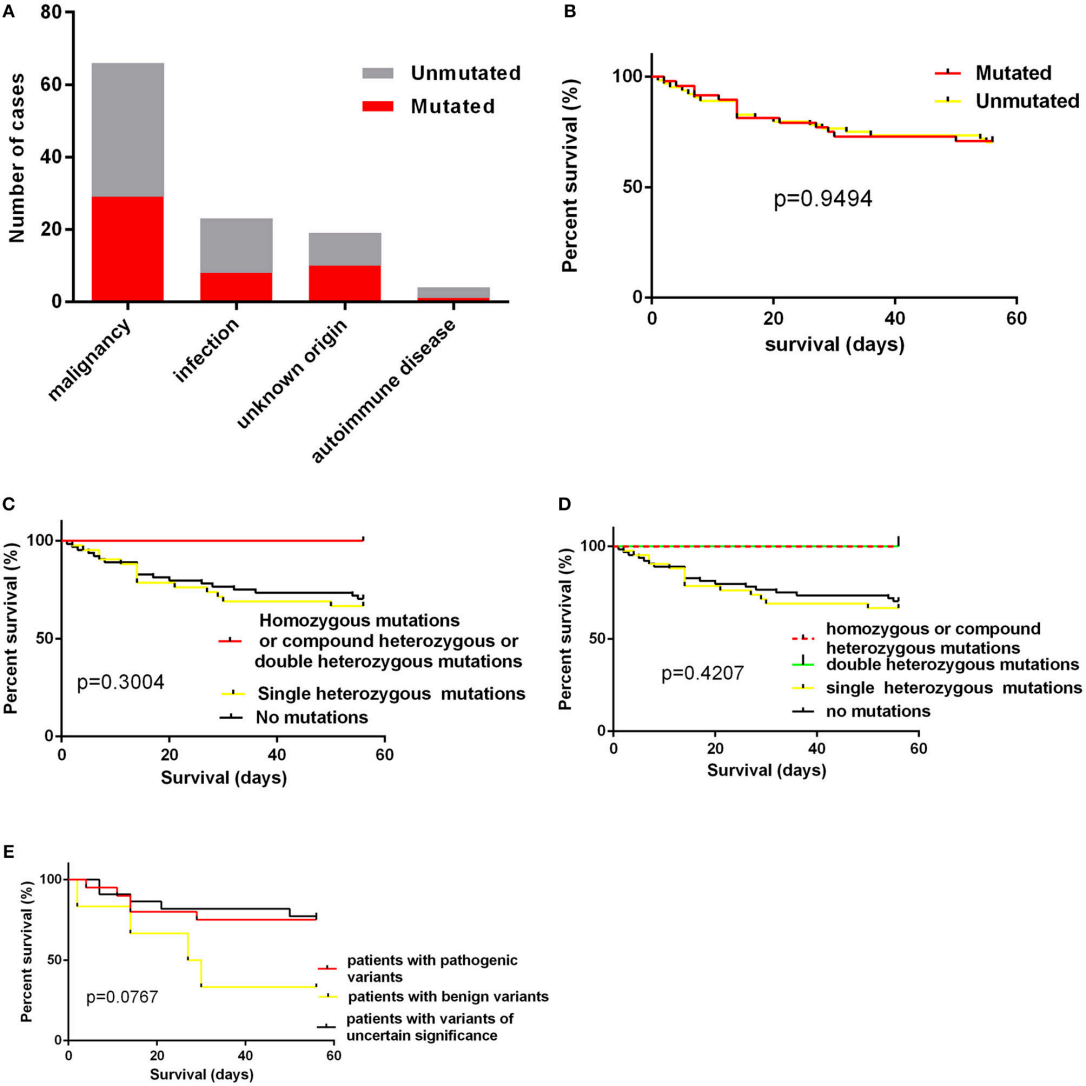
**(A)** the numbers of cases with mutations according to underlying causes; **(B)** the impact of presence of mutations on short-term survival; **(C,D)** the impact of homozygous/compound heterozygous mutations or double heterozygous mutations on short-term survival; **(E)** the short term survival of patients with pathogenic variants, patients with benign variants and patients with variants of uncertain significance.

The age of cases with identified variants was similar to those without identified variants (median: 46.5 years vs. 48 years, *p* = 0.3310). The frequency of cases with mutations or variants did not differ among four groups, which were classified by underlying causes (Figure 3A). For genes mutated in > = 3 cases, the mutation rate of individual gene did not vary among these four groups (Table 3). The frequency of mutation in at least one of these genes involved in the degranulation pathway including *UNC13D, STXBP2, LYST*, and *AP3B1* did not differ significantly among these four groups (Table 3). There was no association between the presence of gene mutations or variants and EBV infection (EBV^+^ 21/52 vs. EBV^−^ 27/60, *p* = 0.7030). However, while considering specific gene mutations, we found there was a trend that cases of EBV infection had more frequent *CORO1A* mutations than the rest of our cohort [3/52 [6.4%] vs. 0/60 [0.0%], OR 8.556, 95% CI: 0.4313–169.7, *p* = 0.0970], which suggested that *CORO1A* mutation may lead to an increased risk of EBV infection.

**Table 3:** Mutation rate of UNC13D, STXBP2, LYST, ITK, CORO1A, and AP3B1 in different groups.

	**Malignancy**	**Infection**	**Unknown origin**	**Autoimmune disease**	***P***
UNC13D	10/66 (15.2%)	0/23 (0%)	2/19 (10.5%)	0/4 (0%)	0.2036
STXBP2	6/66 (9.1%)	1/23 (4.3%)	2/19 (10.5%)	0/4 (0%)	0.7936
LYST	2/66 (3.0%)	3/23 (13.0%)	3/19 (15.8%)	0/4 (0%)	0.1485
ITK	1/66 (1.5%)	2/23 (8.7%)	1/19 (5.3%)	0/4 (0%)	0.4241
CORO1A	2/66 (3.0%)	1/23 (4.3%)	0/19 (0%)	0/4 (0%)	0.8230
AP3B1	3/66 (4.5%)	0/23 (0%)	0/19 (0%)	1/4 (25.0%)	0.0697
At least one of UNC13D, STXBP2, LYST, or AP3B1	21/66	4/23 (17.4%)	7/19 (36.8%)	1/4 (25.0%)	
	(31.8%)				0.5077

We also examined the impact of the presence of gene mutations on short-term survival of HLH patients and found that the detection of HLH-associated gene mutations did not confer a worse short-term prognosis (Figure 3B).The outcome of patients with homozygous or compound heterozygous mutations (*n* = 3) or patients with double heterozygous mutations (*n* = 3) was not significantly different from that of others (Figures 3C,D). And we classified patients with variants or mutations into three groups: patients with pathogenic variants (at least one pathogenic variant) (*n* = 20), patients with benign variants (for cases with two variants, both two variants predicted to be benign) (*n* = 6), and patients with variants of uncertain significance (*n* = 22). The underlying causes did not differ among these three groups. We also found that there was no significant difference in short-term survival among these three groups. There was a trend that patients with benign variants had a worse short-term outcome (Figure 3E, *p* = 0.0767). However, it is hard to draw a conclusion as there were only six patients in this group.

## Discussion

The advent of high-throughput sequencing allows sequencing of hundreds of genes at one time. In the present study, we sequenced 18 HLH-related genes in 112 cases adults HLH patients using TGS. These 18 genes included genes that have been reported to be related to the pathogenesis of HLH as well as those involved in cytotoxicity of NK cells or T cells, which may potentially contribute to the development of HLH. Mutations or rare variants in HLH-related genes were uncovered in cases with or without underlying causes. In contrast to primary HLH, in which homozygous mutations or compound heterozygous mutations were always identified, most of patients in our cohort with mutations only harbored one mutated allele (26). Only one case with homozygous mutation and two cases of compound heterozygous mutations were identified. Three cases with double heterozygous mutations were identified, suggesting mutations of two genes may cooperate in the pathogenesis of HLH. Additionally, almost all the variants were missense mutations in our cohort, which is different from that in pediatric cases in which nonsense and frame-shift mutations are much more prevalent (26), suggesting that most of variants identified in adult HLH may partly impair rather than completely abrogate the function of affected proteins.

In our study, the most frequently mutated gene was *UNC13D*, which was different from a previous study by Wang et al. in which *PRF1* was the most mutated gene (20). A possible explanation for this discrepancy is different populations included in two studies (20). Other reasons could be also possible. Several recurrent gene mutations were identified. The most frequent recurrent variant in our cohort was *UNC13D* p.G863D. Interestingly, homozygous *UNC13D* p.G863D mutation has been previously reported in a male adult HLH patient and his asymptomatic sister also harbored this mutation (27). Both of them displayed impaired NK cell activity and decreased CD107a expression, suggesting the pathogenic role of *UNC13D* p.G863D mutation in HLH (27). *UNC13D* p.G863D mutation is in the MHD2 protein domain of UNC13D, which mediates localization on recycling endosomes and lysosome (28). Homozygous missense *ITK* mutations have been linked to fatal EBV-associated lymphoproliferation (29). In our cohort, *ITK* p.R581W was found to be potentially associated with HLH and *ITK* p.R581W mutation has be reported to be associated with EBV associated T/NK-cell lymphoproliferative diseases and familial HLH (17, 30). However, we did not confirm any significant association between *ITK* p.R581W mutation and EBV infection status in our cohort, suggesting that further functional studies to characterize the biological significance of *ITK* p.R581W is necessary. Another important recurrent variant identified in our cohort is *AP3B1* p.T359A, which has been previously reported in a case of adult hemophagocytic lymphohistiocytosis (16). Although some variants in our cohort are not recurrent, the absence of these variants in normal East Asian population and *in silico* analysis of them suggest the variants are possibly pathogenic in HLH.

Our results suggest that, adult cases with HLH including those with an identified second cause could have possible genetic defects. These genetic defects, mostly heterozygous missense mutations, do not lead to HLH in normal conditions. When a second trigger such as lymphoma occurs, these cases with genetic defects have an increased risk of developing HLH, when compared with those cases without genetic defects. Cases with these mutations show a similar clinical outcome to those without mutations, suggest these mutations may not confer a poor prognosis. Therefore, these heterozygous mutations, which are different from those in pediatric familial HLH cases, could be classified as hypomorphic mutations (3). Regarding therapeutic options, these cases with heterozygous mutations may not need to receive frontline allogenic stem cell transplantation, which are used to treat pediatric cases, since the outcome of these cases are dependent on the underlying causes (31).

As this is a retrospective study, we are not able to conduct immunologic experiments including NK cell cytotoxicity, perforin or CD107a expression tests in our patients, making it less possible for us to analyze the association between those phenotypic characteristics and genetic variants. Although TGS enables simultaneous sequencing of multiple genes, in some cases, it remains insufficient for exploring the mechanisms underlying development of HLH. According to a study in pediatric cases (32), whole-exome sequencing (WES) can help find molecular explanations for the pathogenesis of HLH when target gene sequencing fails. This may also apply to adult cases with HLH.

In conclusion, targeted gene sequencing of HLH-related genes in adult HLH patients revealed a possible role of genetic factors in the pathogenesis of adult HLH. Further studies, which include immunologic tests and genetic testing, especially WES, will be helpful in characterizing the role of genetic factors in the development of adult HLH.

## Author Contributions

J-YL and WX designed and supervised the study. YM, H-YZ, J-YL, and WX analyzed the data and drafted the manuscript. YX, YK, Y-XZ, Y-QM, XC, CQ, and LC performed the experiments and collected the data. J-HL, J-ZW, LW, WW, and LF collected the clinical data. All authors read and approved the final manuscript.

### Conflict of Interest Statement

The authors declare that the research was conducted in the absence of any commercial or financial relationships that could be construed as a potential conflict of interest.
